# Case Report: Multi-sensory integration treatment for preschool children with intermittent exotropia and idiopathic scoliosis comorbidity

**DOI:** 10.3389/fmed.2026.1735918

**Published:** 2026-02-02

**Authors:** Yu Huang, Zheng Li, Fanling Zeng, Yashu Li, Xidan Deng, Cheng Yang, Jin Zeng, Yanlei Chen

**Affiliations:** 1 Department of Ophthalmology, Guangdong Provincial People’s Hospital, Guangdong Academy of Medical Sciences, Southern Medical University, Guangzhou, Guangdong, China; 2 Department of Ophthalmology, The Second People’s Hospital of Foshan, Guangdong Pharmaceutical University, Foshan, Guangdong, China; 3 Department of Rehabilitation, Guangdong Provincial People’s Hospital, Guangdong Academy of Medical Sciences, Southern Medical University, Guangzhou, Guangdong, China; 4 Department of Rehabilitation Medicine, The First Affiliated Hospital, Sun Yat-sen University, Guangzhou, Guangdong, China; 5 National Engineering Research Center for Healthcare Devices, Guangzhou, Guangdong, China

**Keywords:** idiopathic scoliosis, intermittent exotropia, multi-sensory integration training (MSIT), postural control, strabismus

## Abstract

This case report evaluates a novel multi-sensory integration training (MSIT) protocol, combining binocular disparate vision perception training with physiotherapeutic scoliosis-specific exercises in two pediatric patients (aged 4 and 5 years) with comorbid non-syndromic strabismus and idiopathic scoliosis. Both patients presented with intermittent exotropia, amblyopia, and thoracic scoliosis (Cobb angles of 10° and 13°, respectively). Following the individualized MSIT intervention, marked improvements were observed in both visual and spinal parameters: visual acuity of the amblyopic eye improved, exotropia magnitude decreased, stereopsis recovered, and Cobb angles substantially reduced to 3° and 0°, respectively, after 3 months. A transient “treatment plateau” in perceptual eye position metrics was observed in one case before subsequent normalization. These findings suggest that MSIT, by simultaneously addressing aberrant visual and proprioceptive inputs, effectively improves both visual function and spinal alignment in this comorbid population, potentially through promoting central nervous system recalibration of multi-sensory integration for postural control. This integrated approach highlights the importance of interdisciplinary management for children presenting with both strabismus and spinal deformities.

## Introduction

Strabismus is a common ocular condition in children and adolescents (1). Recently, newly developed binocular disparate vision perception training has become popular among children who are ineligible or unwilling to undergo surgery (2, 3). However, unsatisfactory training outcomes, including protracted treatment durations or relapse upon cessation of training, have been observed in certain patients. Therefore, elucidating the underlying factors contributing to these unsatisfactory treatment results may assist in the development of better intervention strategies.

Similar to strabismus, idiopathic scoliosis (IS), which is characterized by spinal deformity without any specific underlying cause, is prevalent among children. The diagnosis is based on a Cobb angle exceeding 10° as determined by a standing full-spine X-ray in the frontal plane (4). Early diagnosis and timely interventions for IS minimize the need for surgery (5). Thus, identifying high-risk individuals would improve the screening efficiency and diagnostic accuracy of IS.

Recent studies have demonstrated that comorbid strabismus and scoliosis can be found in children without severe congenital syndromic diseases, such as Angelman Syndrome and Prader–Willi Syndrome (6, 7). It has been noted that the prevalence of IS is considerably higher among children with strabismus than among those without the condition (8, 9). However, the pathogenesis of this comorbidity currently lacks specific treatment strategies for non-syndromic strabismus and IS.

This study reported two cases of children with non-syndromic strabismus and IS, and focuses on the effect of a proposed therapeutic strategy, namely “Multi-sensory Integration Training (MSIT),” which combines vision perception training with physiotherapeutic scoliosis-specific exercises. Our findings indicate that this treatment is associated with improvement in exotropia, reduction in Cobb angle, shorter treatment duration, and a stable therapeutic effect.

## Case presentation

### Case 1

**Figure 1:**
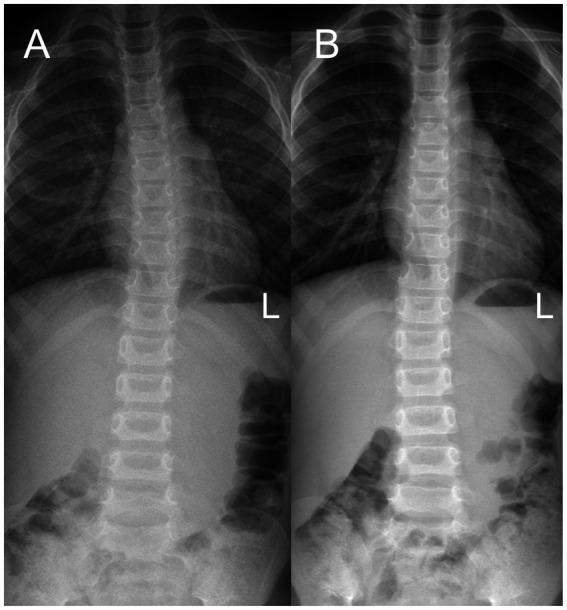
Full-length spinal anteroposterior X-ray radiography in case 1. **(A)** The Cobb angle was 10° before treatment. **(B)** The Cobb angle was 3° after 3 months of MSIT treatment.

A 4-year-old boy presented to our hospital with a 6-month history of frequent outward deviation of both eyes. He was born at term with normal birth parameters and demonstrated normal growth and developmental milestones. On examination, a refractive error of +1.25DS in the right eye and 0 D in the left eye was found by cycloplegic refraction. The Snellen best-corrected visual acuity (BCVA) was 20/50 in the right eye and 20/20 in the left eye. Hirschberg test results were negative at the primary position near the gaze. An alternate cover test found approximately 15-degree exotropia near and orthophoria at a distance. No nystagmus or ptosis was observed. Perceptual eye position (PEP) and third-order stereopsis function (zero-, first-, and second-order) were measured as previously described, and the Worth 4 dot test was abnormal (3). A visual and perceptual examination evaluation system invented by the National Engineering Research Center for Healthcare Devices was used. Details of the eye examinations are shown in Table 1. We also noticed that his left shoulder was higher than the others when standing; hence, he was referred to the department of rehabilitation. The Adams forward bending test was positive, and the truck rotation angle (ATR) was 4° at the 12th thoracic vertebrae. Full-length spinal anteroposterior and lateral radiographs showed that the Cobb angle of the ninth thoracic scoliosis curvature was 10° (Figure 1A).

**Table 1:** Clinical characteristics of the patient in case 1.

Characteristics		Baseline	1 month	3 months	4 months	6 months
BCVA	Right eye	20/50	20/32	20/25	20/20	20/20
	Left eye	20/20	20/20	20/20	20/20	20/20
Deviation degree		−15		−5		−3
PEP	Horizontal	425	206	706	41	19
	Vertical	15	3	89	2	2
Stereopsis	S0	0	1	1	1	1
	S1	1	1	1	1	1
	S2	1	1	1	1	1
Cobb angle		10	–	3	–	3

BCVA, best-corrected visual acuity; PEP, perceptual eye position.

S0 (zero-order stereopsis) was graded as follows: 0 = failed test, 1 = 100 arcseconds, 2 = 200 arcseconds, 3 = 400 arcseconds, 4 = 800 arcseconds.

S1 (first-order stereopsis) was graded as follows: 0 = failed test, 1 = passing at a speed of 50 arcs/ms, and 2 = passing at a speed of 600 arcs/ms.

S2 (second-order stereopsis) was graded as follows: 0 = failed test, 1 = 100% accuracy, 2 = 75% accuracy, 3 = 50% accuracy, 4 = 25% accuracy.

PEP and stereopsis were assessed with a visual and perceptual evaluation system.

The diagnosis was intermittent exotropia and amblyopia of the right eye by the Department of Ophthalmology and mild IS by the Department of Rehabilitation. The patient received an individualized therapeutic regimen for the MSIT. Ophthalmic interventions included spectacles (right eye of +0.50DS) and individualized binocular disparate vision perception training prescribed by ophthalmologists, while rehabilitation interventions included individualized physiotherapy scoliosis-specific exercises (PSSE) prescribed by rehabilitation therapists. Binocular disparate vision perception training was delivered via software provided by the National Engineering Research Center for Healthcare Devices. The protocol was customized based on each patient’s binocular visual function assessment and administered using a video terminal combined with red-blue glasses/VR head-mounted device technology (3). The PSSE component consisted of standard, conventional physiotherapeutic regimens prescribed by the rehabilitation department, comprising a set of tailored physical movements to be performed daily by the patient (e.g., cat breathing and double hip joint swing).

One month later, the BCVA was 20/32 in the right eye. The zero-order stereoscopic function and the horizontal deviation of the PEP were improved. Three months later, during the routine follow-up of PSSE, marked improvement in thoracic scoliosis was observed, and X-ray radiography revealed a Cobb angle of 3° (Figure 1B). During the visit to the ophthalmologist, the parents reported an obviously reduced frequency of outward deviation from the eyes. The BCVA was 20/25 in the right eye, and the Worth 4 dot test found no suppression. An alternate cover test showed 5-degree exotropia near and orthophoria at distance. However, the PEP results showed greater deviation in the horizontal and vertical eye positions compared to the initial visit. Because the overall condition was ameliorated, the original therapy regimens were administered. Four months later, the BCVA was 20/20 in both eyes; interestingly, there was a large improvement in horizontal and vertical deviation of PEP compared with the condition on the third ophthalmology visit. Six months later, the BCVA and PEP in both eyes remained stable (Table 1). The patient has been followed every 3 months since then, with both visual acuity and PEP remaining improved up to the time of documentation of this case report.

### Case 2

**Figure 2:**
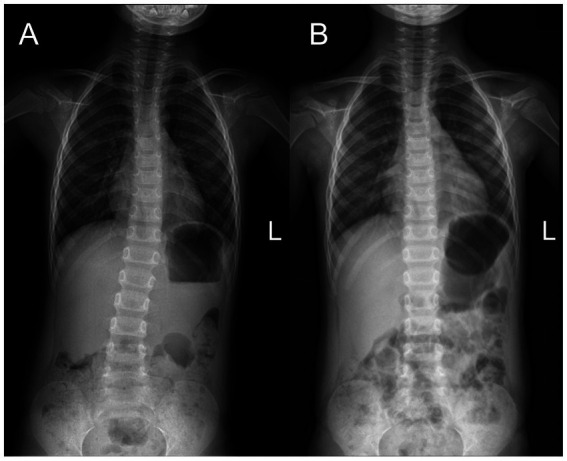
Full-length spinal anteroposterior X-ray radiography in case 2. **(A)** The Cobb angle was 13° before treatment. **(B)** The Cobb angle was 0° after 3 months of MSIT treatment.

A 5-year-old boy presented with intermittent exotropia and amblyopia in the right eye. He was also born full-term with no anomalies during pregnancy, delivery, or developmental milestones. Post-dilation refractive examination revealed a refractive error of +4.50DS/−0.50 DC in the right eye, corrected to 20/50, and +2.25DS/−0.50 DC in the left eye, corrected to 20/30. The Hirschberg test was negative, and the alternate cover test showed approximately 30 degrees of exotropia. The ocular motility examination was normal, fundus examination revealed no abnormalities, and the Bielschowsky test was negative. Visual function tests indicated central suppression and abnormal perceived eye position in the right eye (Table 2). The patient’s posture was abnormal while standing, with the head tilted to the right and left shoulders elevated compared to the right, and the chest rotated to the right. The Adams forward bend test results were positive. Spinal radiography revealed mild scoliosis at the 11th thoracic vertebra with a Cobb angle of 13° (Figure 2A). The patient was diagnosed with exotropia and IS, and MSIT, including corrective glasses, visual perceptual training, and PSSE, were prescribed.

**Table 2:** Clinical characteristics of the patient in case 2.

Characteristics		Baseline	1 month	3 months	6 months
BCVA	Right eye	20/50	20/32	20/30	20/25
	Left eye	20/30	20/25	20/25	20/20
Deviation degree		−30		−10	−5
PEP	Horizontal	34	5	24	13
	Vertical	5	8	8	4
Stereopsis	S0	0	0	1	1
	S1	0	0	1	1
	S2	1	1	1	1
Cobb angle		13	–	0	0

BCVA, best-corrected visual acuity; PEP, perceptual eye position.

S0 (zero-order stereopsis) was graded as follows: 0 = failed test, 1 = 100 arcseconds, 2 = 200 arcseconds, 3 = 400 arcseconds, 4 = 800 arcseconds.

S1 (first-order stereopsis) was graded as follows: 0 = failed test, 1 = passing at a speed of 50 arcs/ms, and 2 = passing at a speed of 600 arcs/ms.

S2 (second-order stereopsis) was graded as follows: 0 = failed test, 1 = 100% accuracy, 2 = 75% accuracy, 3 = 50% accuracy, 4 = 25% accuracy.

PEP and stereopsis were assessed with a visual and perceptual evaluation system.

One month later, the visual acuity of the right and left eyes had improved to 20/32 and 20/25, respectively. A marked improvement in PEP was observed, while stereopsis remained unchanged. Three months after treatment, there was marked improvement in thoracic scoliosis, which was confirmed by radiography (Figure 2B). The parents reported a reduction in the frequency and magnitude of exotropia. Upon examination at the sixth month, the visual acuity of the right eye was 20/25, and that of the left eye was 20/20, with an eye position of 5-degree exotropia and no suppression in the right eye. Third-order stereopsis function was recovered; the Worth 4 dot test found no suppression (Table 2). He continued with initial treatment and had follow-ups every 3 months.

## Discussion

Misalignment of the visual axes of both eyes in individuals with strabismus may be related to musculoskeletal disorders. To mitigate strabismus symptoms and achieve optimal binocular visual function, patients often adjust the positioning of their head or neck, such as tilting or rotating, to reduce ocular misalignment; such ocular causes account for 18 to 25% of cases of anomalous head posture (AHP) (10). IS is another possible type of musculoskeletal disorder in addition to AHP. Scoliosis is often accompanied by postural abnormalities, including head displacement, trunk curvature, and unstable standing, which are manifested in quantitative measures of postural stability, such as larger variability in the center of pressure and sway area (4, 11). Similar change patterns in the measurement outcomes of posture control and marked visual discrepancies have been observed in children with strabismus (12–14).

The potential pathogenesis of strabismus and IS may be related to postural control, which involves the integration of sensory inputs from multiple systems, complex information processing, motor output, and real-time adjustments (15–18). Any dysfunction of the visual, vestibular, or proprioceptive systems may prompt individuals to recalibrate the weighting of sensory inputs from different sensory systems to compensate for erroneous sensory information (12, 13, 17). Strabismus may cause abnormal visual input, affecting the integration and coordination of the proprioceptive system, and vice versa (13, 14). Prolonged erroneous visual/proprioceptive inputs may lead to corresponding postural compensations at the outset and progress into the establishment of an incorrect balance of motor control in central nervous system and musculoskeletal system disorders such as IS (14–16).

In our patient, MSIT was associated with a marked improvement in both binocular visual function and spinal morphology. Thus, for patients with strabismus and IS comorbidity, we propose that the MSIT is needed until the establishment of correct and balanced processing and feedback loops in the central nervous system. We emphasize a combined ‘multi-sensory’ intervention, as single-sensory treatments—such as visual perception training alone without ignoring the PSSE—may fail to address the erroneous sensory input from the body musculoskeletal system; consequently, maladaptive processing patterns may persist within the central nervous system. These may be potential reasons for suboptimal outcomes or recurrence of strabismus or IS.

Although scoliosis showed marked improvement by the third month, the patient’s PEP deteriorated compared with the initial diagnosis. However, both the degree of strabismus and the visual acuity of the amblyopic eye showed evident improvement. Interestingly, at the fourth month, the patient’s PEP level had returned to normal. This type of phenomenon, where the clinical condition appears to improve while quantitative indicators show deterioration or plateauing, or fluctuations between improvement and relapse, can be referred to as “treatment plateau.” We hypothesized that this may stem from the reweighting of the central nervous system and the coordination of visual and proprioceptive inputs. When new or corrected sensory inputs are established, the central nervous system needs to adjust the original processing patterns, re-weight, and adapt to the new one, which may require different times and effort for different individuals (13, 17).

Currently, the regulation of this adjustment remains unclear, but it is evident that continued intervention with MSIT is necessary when the treatment reaches a plateau phase. However, the duration of this adjustment appeared to vary among patients and age groups. The early years of human life are critical for visual development. In patients with strabismus, misalignment of the visual axes causes visual perception issues with an earlier onset, potentially causing amblyopia and faulty visual input (19). Nonetheless, this stage also represents the period of greatest neural plasticity of the central nervous system (16, 19). Therefore, timely treatment might help reshape the synaptic connections between neural networks involved in visual perceptual processing and other systems involved in posture control, restoring normal binocular relationships and postural stability.

According to the 2016 SOSORT guidelines, a Cobb angle greater than 10° on standing X-rays is required for a diagnosis of scoliosis (4). Following this definition, studies by Pan et al. (9) and Zhu et al. (8) determined that post-strabismus surgery children had a higher thoracic scoliosis incidence compared to their normal peers. However, for those with Cobb angles under 10° but exhibiting spinal curve deformities, the mainstream approach is periodic observation (4). Cobb angle measurements may be influenced by factors such as equipment errors, subjective selections, and suboptimal cooperation from children, leading to approximately 5° of potential error, and as a result, some children are possibly misclassified as normal by measurement errors, delaying diagnosis and treatment of IS (4, 20). Hence, we propose a new concept called “latent idiopathic scoliosis (LIS),” which refers to cases with spinal curve deformities (on standing X-rays) but with Cobb angles below 10°, accompanied by visual abnormalities, including strabismus or AHP. In patients with LIS, improper visual, neuromuscular, and proprioceptive inputs may intensify the severity of strabismus and IS, making treatment more challenging. Thus, early MSIT intervention measures may be necessary to prevent and treat the development of strabismus and IS, beyond the standard periodic observation approach.

Our case report suggests a potential link between strabismus and IS, with patients with strabismus possibly being at risk of concurrent IS. We recommend that when pediatricians detect strabismus during routine checkups, further spinal evaluations should be considered to screen for potential IS. The MSIT, which combines strabismus treatment with PSSE, highlights the need for a multi-sensory integration approach to normalize posture control by the central nervous system. Moreover, our findings highlight the need for an interdisciplinary framework for strabismus based on posture control theory. This framework requires the collaboration of multiple subspecialties, including pediatric geneticists, pediatric growth and development specialists, strabismus and amblyopia specialists, optometrists, rehabilitation physicians, sports medicine doctors, and radiologists. By developing standardized treatment procedures, we aim to enhance treatment outcomes, reduce treatment duration, and lower recurrence rates by prioritizing non-surgical interventions.

## Data Availability

The original contributions presented in the study are included in the article/supplementary material, further inquiries can be directed to the corresponding authors.

## References

[1] Sun Y Zhu B Li L Li H Qiu Y Wang S . Prevalence of visual impairment and refractive error-related risk factors in preschool children in Beijing, China. BMC Public Health. (2025) 25:2433. doi: 10.1186/s12889-025-23485-7, 40646485 PMC12247378

[2] Chan HS Tang YM Do CW Ho Yin Wong H Chan LY To S. Design and assessment of amblyopia, strabismus, and myopia treatment and vision training using virtual reality. Digit Health. (2023) 9:20552076231176638. doi: 10.1177/20552076231176638, 37312939 PMC10259136

[3] Li X Yang C Zhang G Zhang Y Lan J Chu H . Intermittent exotropia treatment with dichoptic visual training using a unique virtual reality platform. Cyberpsychol Behav Soc Netw. (2019) 22:22–30. doi: 10.1089/cyber.2018.0259, 30457355

[4] Negrini S Donzelli S Aulisa AG Czaprowski D Schreiber S de Mauroy JC . SOSORT guidelines: orthopaedic and rehabilitation treatment of idiopathic scoliosis during growth. Scoliosis Spinal Disord. (2016) 13:3. doi: 10.1186/s13013-017-0145-8 PMC5795289 29435499

[5] Dunn J Henrikson NB Morrison CC Blasi PR Nguyen M Lin JS. Screening for adolescent idiopathic scoliosis: evidence report and systematic review for the US preventive services task force. JAMA. (2018) 319:173–87. doi: 10.1001/jama.2017.11669, 29318283 PMC13480065

[6] Basak S Basak A. Proteins and proteases of Prader-Willi syndrome: a comprehensive review and perspectives. Biosci Rep. (2022) 42:BSR20220610. doi: 10.1042/BSR20220610, 35621394 PMC9208313

[7] Gallego-Siles JR Siles-Fuentes MJ Ibáñez-Vera AJ Cortés-Pérez I Obrero-Gaitán E Lomas-Vega R. Idiopathic scoliosis in subjects with eye diseases: a systematic review with meta-analysis. Ann N Y Acad Sci. (2024) 1533:81–8. doi: 10.1111/nyas.15102, 38327125

[8] Zhu B Wang X Fu L Yan J. Pattern strabismus in a tertiary hospital in southern China: a retrospective review. Medicina. (2022) 58:1018. doi: 10.3390/medicina58081018, 36013485 PMC9414984

[9] Pan XX Huang CA Lin JL Zhang ZJ Shi YF Chen BD . Prevalence of the thoracic scoliosis in children and adolescents candidates for strabismus surgery: results from a 1935-patient cross-sectional study in China. Eur Spine J. (2020) 29:786–93. doi: 10.1007/s00586-020-06341-7, 32112152

[10] Akbari MR Khorrami-Nejad M Kangari H Akbarzadeh Baghban A Ranjbar PM. Ocular abnormal head posture: a literature review. J Curr Ophthalmol. (2021) 33:379–87. doi: 10.4103/joco.joco_114_20, 35128182 PMC8772496

[11] Wen JX Yang HH Han SM Cao L Wu HZ Yang C . Trunk balance, head posture and plantar pressure in adolescent idiopathic scoliosis. Front Pediatr. (2022) 10:979816. doi: 10.3389/fped.2022.979816, 36340704 PMC9627203

[12] Reche-Sainz JA Ruiz-Aimituma F Toledano-Fernández N. Comparison of postural control between strabismic and non-strabismic children. Arch Soc Esp Oftalmol. (2021) 96:10–8. doi: 10.1016/j.oftal.2020.06.008, 32690373

[13] Jayakaran P Aman W Fernando U Hackfath K McPherson A Williams M . Sensory organization for postural control in children with strabismus-a systematic review and meta-analysis. Gait Posture. (2021) 88:94–104. doi: 10.1016/j.gaitpost.2021.05.008, 34015547

[14] Papalia GF Mangano G Diaz Balzani LA Cupo G Giurazza G Di Zazzo A . Strabismus and postural control: a systematic review. Musculoskelet Surg. (2022) 106:345–56. doi: 10.1007/s12306-022-00737-y, 35187611

[15] Luo H Wang X Fan M Deng L Jian C Wei M . The effect of visual stimuli on stability and complexity of postural control. Front Neurol. (2018) 9:48. doi: 10.3389/fneur.2018.00048, 29472888 PMC5809403

[16] Takakusaki K. Functional neuroanatomy for posture and gait control. J Mov Disord. (2017) 10:1–17. doi: 10.14802/jmd.16062, 28122432 PMC5288669

[17] Morningstar MW Pettibon BR Schlappi H Schlappi M Ireland TV. Reflex control of the spine and posture: a review of the literature from a chiropractic perspective. Chiropr Osteopat. (2005) 13:16. doi: 10.1186/1746-1340-13-16, 16091134 PMC1198239

[18] Sinno S Dumas G Mallinson A Najem F Abouchacra KS Nashner L . Changes in the sensory weighting strategies in balance control throughout maturation in children. J Am Acad Audiol. (2021) 32:122–36. doi: 10.1055/s-0040-1718706, 33296934

[19] Milleret C Bui Quoc E. Beyond rehabilitation of acuity, ocular alignment, and binocularity in infantile strabismus. Front Syst Neurosci. (2018) 12:29. doi: 10.3389/fnsys.2018.00029, 30072876 PMC6058758

[20] Jin C Wang S Yang G Li E Liang Z. A review of the methods on cobb angle measurements for spinal curvature. Sensors (Basel). (2022) 22:3258. doi: 10.3390/s22093258, 35590951 PMC9101880

